# Dr. J. N. Farrar's Lectures

**Published:** 1880-01

**Authors:** 


*Lectures.*—Dr. J. N. Farrar, of Brooklyn, gave during the second week in December a course of lectures to the Students of the Baltimore College of Dental Surgery, on "The Direct Process in Regulating Teeth," elucidating the prominent parts in the method advocated by him, and illustrating by models and apparatus the advantages of the same. A report of the lectures is promised soon.

